# Silane cooperation with Ce_2_(SO_4_)_3_ to efficiently construct a protective layer and induce uniform deposition of Zn^2+^ for an ultra-stable Zn anode†

**DOI:** 10.1039/d5sc00718f

**Published:** 2025-03-26

**Authors:** Luyan Yu, Sidan He, Baohua Liu, Mingrui Zhang, Houyi Ma, Chao Wang, Qinghong Wang

**Affiliations:** a School of Chemistry and Materials Science, Jiangsu Normal University Xuzhou Jiangsu 221116 P. R. China wangc@jsnu.edu.cn wangqh@jsnu.edu.cn; b School of Chemistry and Chemical Engineering, Shandong University Jinan Shandong 250100 P.R. China

## Abstract

Aqueous Zn ion batteries (ZIBs) are gaining interest for use in large-scale energy storage systems due to their intrinsic safety, low cost, and sustainability. Unfortunately, water-induced side reactions and dendrite growth on the Zn anode have severely hampered their further development. Herein, 3-aminopropyltrimethoxysilane (KH-540) was employed as an electrolyte additive to construct an organic–inorganic solid-electrolyte interface (SEI) layer comprising zinc hydroxy sulfate and polysiloxane to alleviate the side reactions and inhibit dendrite growth. Moreover, Ce_2_(SO_4_)_3_ was simultaneously introduced to shield the protuberances on the surface of the Zn anode, thus effectively decreasing the “tip effect”. Due to the synergistic effect of the dual additives, uniform Zn deposition was achieved and the cycling stability of Zn anode significantly improved. As a result, the Zn‖Zn symmetric battery exhibits a long cycle life of 5000 h at a current density of 1.0 mA cm^−2^ with an areal capacity of 1.0 mA h cm^−2^, as well as high Coulombic efficiency of nearly 100%. The Zn‖V_2_O_5_ full cell delivers a high specific capacity of 188.35 mA h g^−1^ even after 1000 cycles.

## Abstract 2

An organic–inorganic solid/electrolyte interface is constructed *in situ* with composite additives to simultaneously induce uniform zinc deposition and suppress side reactions, further improving the electrochemical performance of zinc ion batteries.

## Graphical abstract

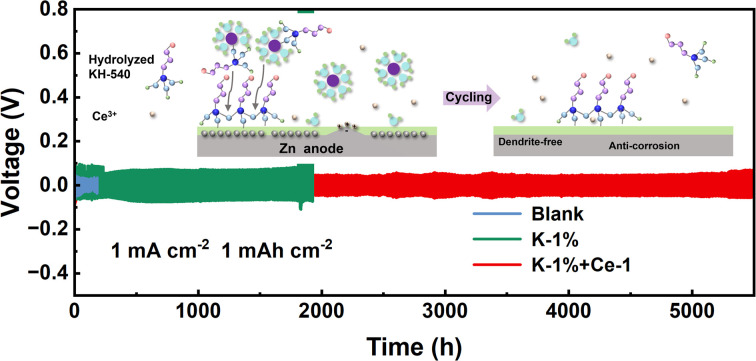

## Introduction

The escalating global demand for renewable energy is driving the development of large-scale energy storage systems.^1^ Safety issues, such as fire and explosion hazards associated with organic electrolyte-based lithium-ion batteries (LIBs), are propelling research toward more secure, sustainable rechargeable battery technologies.^2,3^ In this context, Zn-ion batteries (ZIBs) have garnered significant interest due to their intrinsic safety features, coupled with the low redox potential (−0.76 V *vs.* SHE) and high theoretical capacity (820 mA h g^−1^, 5855 mA h cm^−3^) of the Zn anode.^4,5^ In addition, the ionic conductivity of the aqueous electrolyte is two orders of magnitude higher than that of the organic electrolyte.^6^ However, the hydrogen evolution reaction (HER) and chemical corrosion of the Zn anode during the cyclic charge–discharge process can generate numerous by-products and cause surface passivation of the electrode, leading to rapid capacity fading and poor Coulombic efficiency (CE).^7–9^ Furthermore, uncontrollable dendrite growth on the Zn anode caused by the “tip effect” can easily pierce the separator and even short circuit the cell.^10,11^

The electrochemical reactions mainly occur at the solid-electrolyte interface (SEI) of the Zn anode.^12^ Constructing a robust protective SEI layer is regarded as an efficient approach, as it precludes water penetration into the Zn anode.^13–16^ However, it is difficult for artificially constructed SEI layers to adapt to the huge volume change of the Zn anode during reversible cycling.^17^ In contrast, *in situ* construction of an SEI layer by employing an electrolyte additive is a relatively simple yet effective strategy due to the multifunctional features: first, inducing homogeneous nucleation on the surface of the Zn anode at the initial plating stage and inhibiting the “tip effect” in the subsequent stage;^18,19^ Second, regulating the interfacial electrostatic field and preventing local Zn deposition;^20,21^ Third, regulating the deposition orientation by changing the solvation structure of Zn^2+^.^22^ However, a single electrolyte additive can rarely satisfy all requirements of an ultra-stable Zn anode.

Recently, composite additives have drawn researchers' attention. The combination of xylitol and graphene oxide (GO) additives not only significantly regulates the solvation structure of Zn^2+^, but also effectively accelerates the reaction kinetics due to the protective effect of the self-assembled GO layer.^23^ Liu *et al.*^24^ found that the co-addition of polyvinyl alcohol (PVA) and vanillin realized the complementary functions of stabilizing the structure of the Zn anode and enhancing the rate capability, thus significantly improving the cycle life and rate performance of the ZIBs. Dai *et al.*^25^ reported a bifunctional additive consisting of ethylene glycol (EG) and sodium gluconate (Ga). The hydroxyl structures of EG and Ga can participate in the hydrogen-bonding network of H_2_O, thereby optimizing the coordination environment around hydrated Zn^2+^ and reduces side reactions. Moreover, Ga in the system can form a stable protective layer and lead to uniform Zn deposition.

**Fig. 1:**
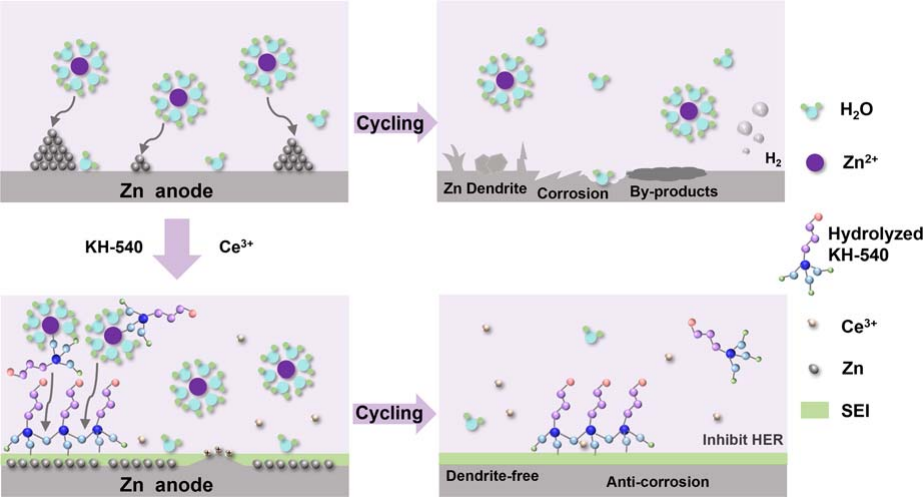
Schematic illustration of the Zn plating in 2 M ZnSO_4_ with and without KH-540 and Ce_2_(SO_4_)_3_.

Silane coupling agents are widely employed as surface modifiers^26,27^ due to the exceptional metal affinity, structural flexibility, and adjustability enabled by abundant functional groups.^28,29^ Metal ions have been proved to be effective in regulating interfacial electric fields.^30,31^ Herein, we propose a dual additive composed of amino silane (KH-540) and Ce_2_(SO_4_)_3_ to induce uniform Zn deposition. As shown in Fig. 1, an organic–inorganic hybrid SEI composed of zinc hydroxyl sulfate and silanol is *in situ* formed on the surface of Zn anode, which not only protects the Zn anode against chemical corrosion, but also provides abundant ion transport channels for uniform Zn deposition. Furthermore, Ce^3+^ acts as a leveling agent to inhibit the “tip effect” during the deposition process. Due to the above synergistic effects, the Zn anodes achieve high reversibility (5000 h at a current density of 1.0 mA cm^−2^ with an areal capacity of 1.0 mA h cm^−2^) and a high CE of 99.49%. Moreover, when matched with a V_2_O_5_ cathode, the full cell employing the composite additive achieves a high capacity retention rate of 78.7% after 1000 cycles at a current density of 2.0 A g^−1^. This work provides a useful approach for the development of efficient electrolyte additives toward high-performance ZIBs.

## Results and discussion

### Formation and characterization of the protective film formed *in situ* by the dual additives

To *in situ* construct a stable SEI layer on the surface of the Zn anode, KH-540 is employed as an electrolyte additive. As shown in Fig. S1,† a double hydrolysis reaction is initiated once KH-540 is added to 2 M ZnSO_4_ solution due to the strong protophilicity of –NH_2_ and –Si–O–CH_3_ functional groups in KH-540 and the electrophilicity of Zn^2+^ in the electrolyte. As a result, the pH value is increased (Fig. S2†), and a suspension containing a white precipitate of 3Zn(OH)_2_·ZnSO_4_·5H_2_O and gelatinous polysiloxane is formed. The generation of inorganic 3Zn(OH)_2_·ZnSO_4_·5H_2_O was confirmed by the X-ray diffraction (XRD) pattern of the precipitate shown in Fig. S3.† The hydrolysis of siloxane in the weakly acidic electrolyte was verified by the disappearance of Si–O–CH_3_ and the appearance of CH_3_–OH peaks in the ^1^H NMR spectra of the K-1% electrolyte compared with KH-540 solution (Fig. S4†). Moreover, the hydrolyzed Si–O–H groups could further crosslink into Si–O–Si networks and form a stable silanol colloid, which can be confirmed by the Tyndall effect (Fig. S5†). The FTIR spectrum of the obtained white precipitate presents obvious signals of S=O, –OH, Zn–OH, and O–S–O bonds from 3Zn(OH)_2_·ZnSO_4_·5H_2_O and Si–O–Si, CH_2_, as well as NH_2_ bonds from silane colloid (Fig. S6†).^29,31–36^ Therefore, a suspension composed of inorganic 3Zn(OH)_2_·ZnSO_4_·5H_2_O and organic polysilane is formed when KH-540 was used as an electrolyte additive, which is expected to *in situ* construct a stable hybrid SEI layer on the surface of the Zn anode. However, the ion conductivity decreases from 48.23 mS cm^−1^ for blank ZnSO_4_ electrolyte to 46.57 mS cm^−1^ for the K-1% electrolyte, and it continues to decrease with the increasing concentration of KH-540 (Fig. S7†). Notably, this conductivity reduction is effectively mitigated when Ce_2_(SO_4_)_3_ is introduced as a co-additive.

**Fig. 2:**
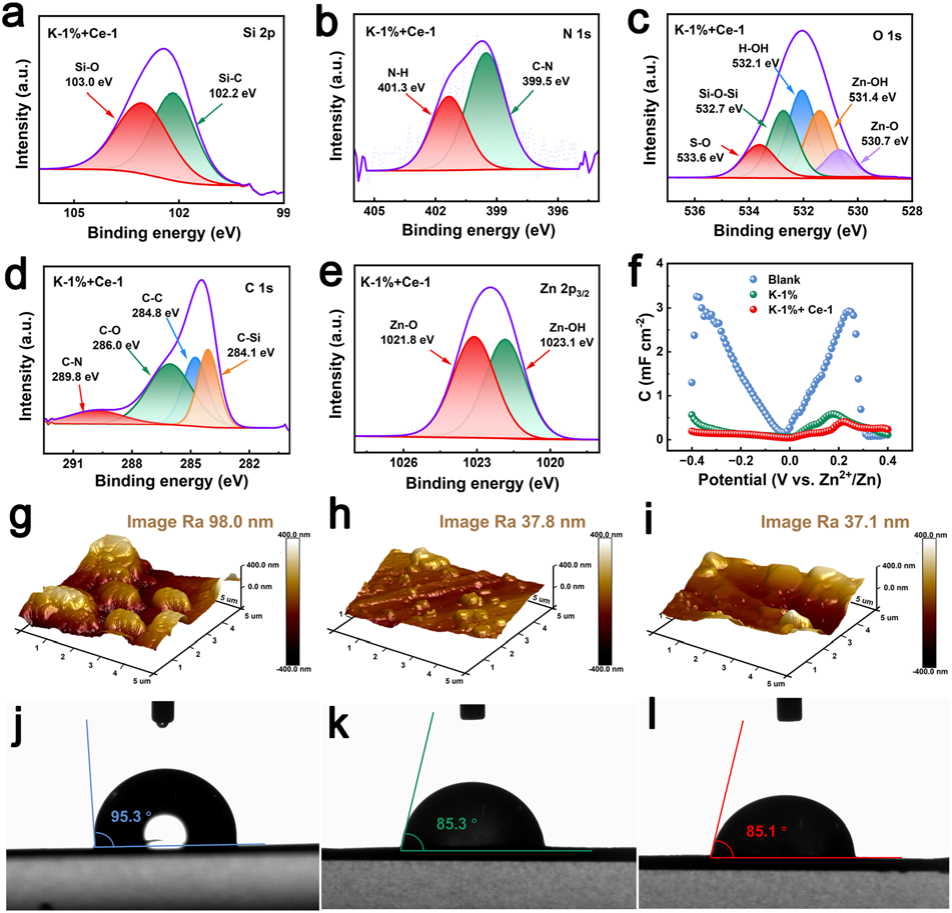
High-resolution XPS spectra of the Zn anode surface after three cycles in K-1% + Ce-1: (a) Si 2p, (b) N 1s, (c) O 1s, (d) C 1s and (e) Zn 2p_3/2_. (f) Differential capacitance curves obtained at 298 K in different electrolyte systems. AFM images of the Zn anodes after three cycles in: (g) blank electrolyte, (h) K-1% and (i) K-1% + Ce-1. Contact angle measurements of the Zn anodes after three cycles in (j) blank electrolyte, (k) K-1% and (l) K-1% + Ce-1.

To confirm the formation of the SEI layer, X-ray photoelectron spectroscopy (XPS) and XRD measurements were performed on the surface of the Zn anode after three cycles in K-1% + Ce-1 electrolyte. As shown in Fig. S8,† the peaks of 3Zn(OH)_2_·ZnSO_4_·5H_2_O were observed in the XRD pattern, and in Fig. S9,† the XPS survey spectrum presents obvious signals of C, Si, N, O, S, Ce, and Zn elements, indicating the formation of the KH-540/3Zn(OH)_2_·ZnSO_4·_·5H_2_O organic–inorganic SEI film on the Zn anode. According to Fig. 2a, the high-resolution XPS spectrum of Si 2p presents characteristic peaks of Si–O and Si–C bonds at 103.0 and 102.2 eV,^37,38^ respectively, indicating the formation of the silanol colloid. In the N 1s fine spectrum (Fig. 2b), the peaks located at 401.3 and 399.5 eV can be attributed to N–H and C–N bonds in K-1% electrolyte, respectively.^39,40^ In the O 1s spectrum (Fig. 2c), S–O (533.6 eV), Si–O–Si (532.7 eV), H–OH (532.1 eV), Zn–OH (531.4 eV), and Zn–O (530.7 eV) bonds were detected.^41,42^ Meanwhile, C–N (289.8 eV), C–O (286.0 eV), C–C (284.8 eV) and C–Si (284.1 eV) bonds were observed in the C 1s spectrum (Fig. 2d) and Zn–O (1021.8 eV) and Zn–OH (1023.1 eV) bonds in the Zn 2p spectrum (Fig. 2e),^43,44^ further demonstrating the generation of the organic–inorganic SEI layer on the surface of the Zn anode. The even distribution of the C, N, Si, O, S, Ce and Zn elements in the EDS mapping of the Zn anode after three cycles (Fig. S10†) further verifies the formation of the hybrid SEI layer. Fig. 2f shows the differential capacitance curves of the Zn anode after three cycles in each electrolyte. It is found that the Zn anode presents much lower adsorption capacitance after cycling in K-1% + Ce-1 and K-1% electrolytes than that in blank electrolyte, indicating that an interfacial protective layer with strong covering ability can be formed on the surface of the Zn anode in the presence of K-1% electrolyte. The AFM images of the Zn anodes after three cycles in K-1% and K-1% + Ce-1 electrolytes present roughness of 37.8 nm and 37.1 nm, respectively, which are significantly lower than that obtained in the blank electrolyte (98.0 nm) (Fig. 2g–i), demonstrating that the *in situ* formed SEI layer could induce uniform Zn deposition, and the addition of Ce_2_(SO_4_)_3_ further enhances the uniformity. Contact angle measurements (Fig. 2j–l) show that the Zn anode after three cycles in blank electrolyte displays hydrophobicity (95.3°), while those present hydrophily after cycling in K-1% (85.3°) and K-1% + Ce-1 (85.1°) electrolytes. The enhanced wettability could be attributed to the abundant functional groups in the *in situ* formed organic–inorganic SEI layer, which will be beneficial for interfacial ion transportation.

### Effects of organic–inorganic hybrid SEI on Zn anode

**Fig. 3:**
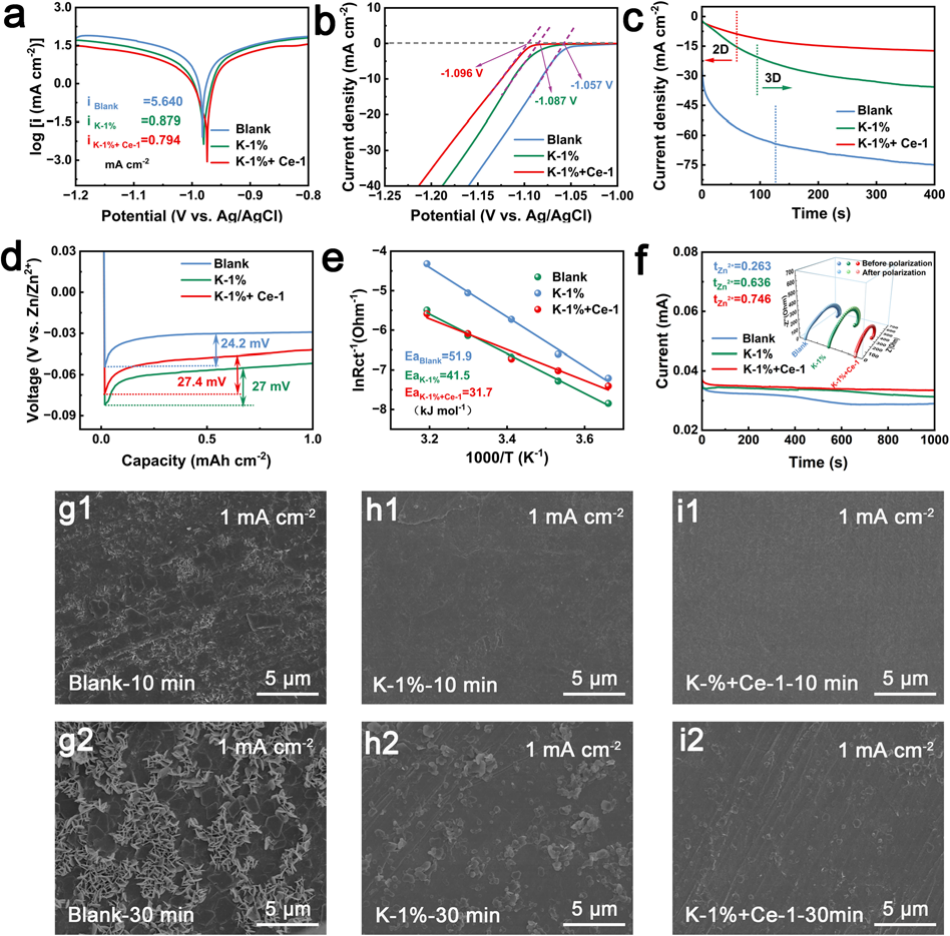
(a) Tafel polarization plots and (b) LSV curves of Zn anodes in each electrolyte. (c) Potentiostatic current–time transient curves at −150 mV. (d) Nucleation overpotential of Zn‖Cu batteries at the first cycle. (e) The calculated desolvation activation energies in different electrolytes. (f) *I*–*t* curve of symmetric cells at an applied voltage of 25 mV. Insets: Nyquist plots before and after polarization. SEM images of the surface morphology after 10 min and 30 min of deposition in (g1 and g2) blank electrolyte, (h1 and h2) K-1% and (i1 and i2) K-1% + Ce-1.

Chemical corrosion on the Zn anode during the charge/discharge process is an important aspect leading to the reduction of CE and battery failure. The effect of KH-540 and Ce^3+^ additives on Zn corrosion was analyzed by Tafel polarization curves. As shown in Fig. 3a and S11,† based on a series of orthogonal tests, when the volume ratio of KH-540 additive was set to 1 vol% and the concentration of Ce_2_(SO_4_)_3_ was set to 1 mM, the Zn anode shows the lowest corrosion current density of 0.794 mA cm^−2^, demonstrating the lowest corrosion rate with the addition of dual additives.^45^ Compared to the blank electrolyte, the corrosion potential of the Zn anode positively shifts from −0.982 V to −0.980 V in K-1% electrolyte, and to −0.974 V in K-1% + Ce-1 electrolyte, indicating less tendency of corrosion reactions with the dual additives.^46,47^ The hydrogen evolution reaction (HER) is another important factor decreasing the cycling stability of Zn anodes because it causes an increase in the local OH^−^ concentration at the electrode/electrolyte interface and yields harmful by-products.^48,49^ To investigate the hydrogen evolution inhibition performance of the Zn anode in the electrolytes with the KH-540 and Ce_2_(SO_4_)_3_ additives, linear scanning voltammetry experiments (LSV) were conducted. As shown in Fig. 3b and S12,† the HER potential shifts negatively in the presence of KH-540 (−1.087 V, *vs.* Ag/AgCl, for the K-1% electrolyte), which becomes more negative with the addition of Ce_2_(SO_4_)_3_ (−1.096 V, *vs.* Ag/AgCl, for the K-1%–Ce-1 electrolyte), further demonstrating that the combined effect of the two additives can inhibit the hydrogen evolution.

The suppressed chemical corrosion and HER in the electrolytes with K-1% and K-1% + Ce-1 electrolytes were further verified by immersing experiments. As shown in Fig. S13a1–3,† vertically oriented flake-like by-products start to form after immersing for 6 h in the blank electrolyte, which become more massive and denser with the extension of the immersion time. In contrast, the Zn anodes present a flat surface without obvious by-products even after immersing for 7 days in K-1% (Fig. S13b1–3†) and K-1% + Ce-1 (Fig. S13c1–3†) electrolytes, showing significantly enhanced anti-corrosion performance. EIS measurements (Fig. S14†) show that the charge transfer resistance of the freshly prepared symmetric cells with the blank electrolyte is smaller than those of the cells with the K-1% and K-1% + Ce-1 electrolyte, which is mainly due to the formation of the inert KH-540/ZnSO_4_·3Zn(OH)_2_·5H_2_O organic–inorganic SEI layer. After 4 h of resting, the charge transfer resistance of the cell with the blank electrolyte significantly increases, which may be due to the formation of a passivation layer caused by chemical corrosion.^50,51^ In contrast, the cells with K-1% and K-1% + Ce-1 electrolytes exhibit a slight increase in charge transfer resistance, indicating that chemical corrosion is effectively alleviated by the as-formed SEI layer, which protects the Zn anode from coming into close contact with the electrolyte.

To study the optimization of Zn^2+^ diffusion on the surface of the Zn anode in the electrolyte with dual additives, Zn nucleation and deposition behaviors were characterized by the chronoamperometry (CA) method at an applied potential of −150 mV. As shown in Fig. 3c, the current density of the Zn anode in K-1% + Ce-1 electrolyte rapidly stabilized during the deposition process, indicating a fast 3D ion migration process after a brief 2D diffusion,^52^ which implies that the diffusion barrier established by the Ce^3+^ and KH-540 layer is effective in homogenizing Zn^2+^ distribution at the interface. In contrast, the current density of the Zn anode in 2 M ZnSO_4_ increased dramatically and could not be stabilized within 200 s, indicating that a vigorous 2D diffusion process is occurring on the surface of the Zn anode and Zn^2+^ tends to diffuse along the surface to energetically favorable tip positions, where aggregation and growth lead to the generation of Zn dendrites.^53^ Moreover, the ion conductivity of the organic–inorganic SEI layer is evaluated to be 3.02 × 10^−5^ S cm^−1^ (Fig. S15†), exhibiting excellent ion transport kinetics.

To determine the effect of Ce^3+^ ion on the deposition of Zn^2+^, DFT calculations were conducted to investigate the charge evolution on the protuberance of the Zn anode after the adsorption of Ce^3+^. As shown in Fig. S16,† the surface of Zn anode is intrinsically electronegative. After the adsorption of Ce^3+^, the local partial charge of Ce–3Zn is positive, which will repel Zn^2+^ from the electrolyte, thus inhibiting the “tip effect”. Therefore, Ce^3+^ is inferred to form a shield layer on the surface of the protuberance and balance the electric field, and induce uniform Zn deposition. Nucleation overpotential serves as a crucial index for assessing the quality of the initial deposition process.^54,55^ In this study, the nucleation overpotential of the Zn anode within diverse electrolytes was investigated by plating/striping measurement at 1 mA cm^−2^. As shown in Fig. 3d, a sharp voltage drop appears in the initial nucleation process, and the nucleation overpotential is calculated to be 24.2 mV in the blank electrolyte. In comparison, the Zn anodes in the K-1% and K-1% + Ce-1 electrolyte present increased overpotentials of 27.0 mV and 27.4 mV, respectively. The higher nucleation overpotential may be attributed to the formation of the inert organic–inorganic interface layer, which will be favorable for the refinement of the grain size and the achievement of more homogeneous Zn^2+^ deposition in the initial Zn deposition stage.

In addition, temperature-dependent EIS measurements were carried out to investigate the kinetics of Zn^2+^ plating/stripping in each electrolyte (Fig. S17†). According to the Arrhenius equation, the activation energy (*E*_a_) of Zn^2+^ transference was calculated to be 31.7 kJ mol^−1^ in K-1% + Ce-1 electrolyte, which is lower than those in K-1% electrolyte (41.5 kJ mol^−1^) and blank electrolyte (51.9 kJ mol^−1^), indicating that the dual additive accelerates the desolvation process of hydrated Zn^2+^ (Fig. 3e). It may be because some of the amino and hydroxy groups in the organic–inorganic layer can participate in the solvation sheath of hydrated Zn^2+^, which could be proved by the ^1^H NMR shown in Fig. S18,† where the ^1^H peak was shifted after the addition of 1% KH-540 to the ZnSO_4_ electrolyte.^56^ The designed K-1% + Ce-1 electrolyte and K-1% electrolyte also yield higher Zn^2+^ migration numbers of 0.746 and 0.636 compared to that of 0.263 obtained in the blank electrolyte (Fig. 3f). The positive effect of the KH-540 and Ce^3+^ additive on the ion migration prove that the as-formed organic–inorganic hybrid membrane could modulate the ion transport process and optimize the Zn plating behavior, which will be favorable for dendrite-free deposition.^57^

To further investigate the Zn deposition behavior, the surficial morphology evolution of the Zn anode during the plating process was visually characterized by SEM. As shown in Fig. 3g1 and 2, a small amount of Zn dendrites appeared on the surface of the Zn anode after 10 min of plating in the blank electrolyte, which gradually evolved into micro-sheets after 30 min. In contrast, in K-1% (Fig. 3h1 and 2) and K-1% + Ce-1 (Fig. 3i1 and 2) electrolytes, dendrite growth can be significantly inhibited. Larger magnification SEM images shown in Fig. S19† further confirm that a dense and flat surface without dendrites was consistently observed throughout the deposition process with the addition of the dual additives, indicating that the combined effect of the as-formed organic–inorganic hybrid film and Ce^3+^ ions effectively inhibited the growth of Zn dendrites.

### Cycling stability of the Zn anode in the electrolyte with dual additives

**Fig. 4:**
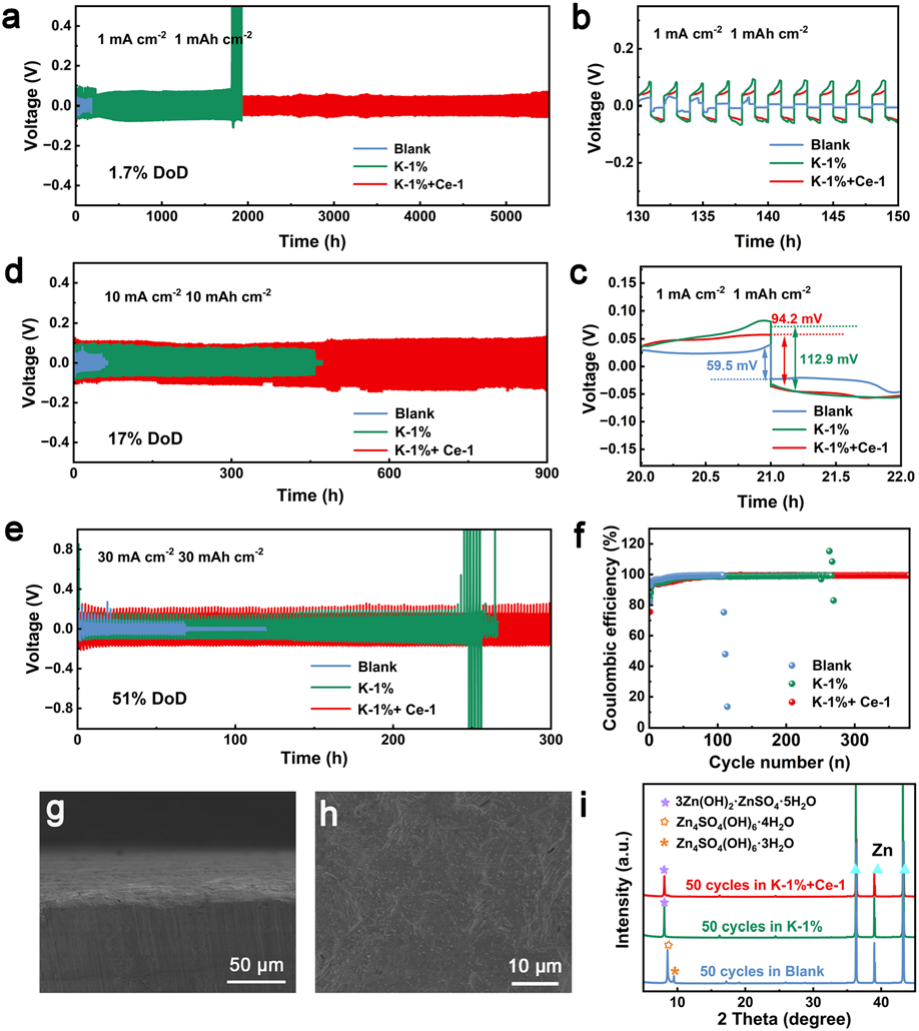
Electrochemical performance of symmetric Zn‖Zn cells: (a) cycling performance at 1.0 mA cm^−2^, 1.0 mA h cm^−2^, and (b and c) the corresponding enlarged galvanostatic charge–discharge curves. Cycling performance at (d) 10 mA cm^−2^, 10 mA h cm^−2^ and (e) 30 mA cm^−2^, 30 mA h cm^−2^. (f) Coulombic efficiency curves of the Zn‖Cu cells at 1 mA cm^−2^. (g) Side-view and (h) top-view SEM images of the Zn anode after 50 cycles at a current density of 1 mA cm^−2^ in K-1% + Ce-1 electrolyte. (i) XRD patterns of the Zn anode after 50 cycles in each electrolyte.

To further evaluate the positive effect of KH-540 and Ce^3+^ additives on optimizing the reversibility of plating/stripping of the Zn anode, Zn‖Zn symmetric cells were assembled and tested. As shown in Fig. S20,† the cells using the electrolyte with dual additives exhibit significantly improved cycling stability (>1400 h) compared to those using a single additive at a current density of 4 mA cm^−2^ with an areal capacity of 4 mA h cm^−2^, confirming the synergistic effect of the additives. It can be confirmed that the optimal electrolyte composition is that containing 1 vol% KH-540 and 1 mM Ce_2_(SO_4_)_3_. To confirm the cycling stability of the Zn anode in the optimized electrolyte, symmetric cells were tested under different current densities. As shown in Fig. S21a† and 4a, the cells with dual additives delivered an ultra long cycle life of 7000 h at a current density of 0.5 mA cm^−2^ with an areal capacity of 0.5 mA h cm^−2^, and 5000 h at a current density of 1.0 mA cm^−2^ with an areal capacity of 1.0 mA h cm^−2^. The cells using the single KH-540 additive also displayed optimized cycling stability which were more durable than those short-circuit early in the blank electrolyte (Fig. 4b). Under the current density of 1 mA cm^−2^ and the areal capacity of 1 mA h cm^−2^, the Zn‖Zn cell with dual additives provides higher nucleation potentials (Fig. 4c), which may be attributed to the generation of inert organic–inorganic SEI layer. Moreover, Zn‖Zn cells keep a steady cycling performance over 900 h under the test conditions of 10 mA cm^−2^, 10 mA h cm^−2^ (Fig. 4d). Depth of discharge (DoD) is an important indicator for evaluating the electrochemical performance of the Zn anode. When the DoD of the Zn anode was set to 34% (20 mA cm^−2^, 20 mA h cm^−2^) and 51% (30 mA cm^−2^, 30 mA h cm^−2^), the cells using the electrolyte with dual additives displayed prolonged cycle lives of 350 h (Fig. S21b†) and 300 h (Fig. 4e), respectively, which are comparable or superior to those of recently reported Zn anodes (Table S1†).

CE is an important parameter for assessing the reversibility and potential application of electrolytes.^58^ The CE of the Zn anodes in each electrolyte was investigated using Zn‖Cu half-cells. As shown in Fig. 4f, the cells using K-1% + Ce-1 electrolyte display the highest CE of 99.49% after 350 cycles at a current density of 1 mA cm^−2^ with an areal capacity of 1 mA h cm^−2^, and the CE of the cells in K-1% electrolyte reached 99.23% after 250 cycles, which are much higher than the 98.21% obtained after 50 cycles in blank electrolyte. The significantly improved CE can be attributed to the inhibited dendrite growth and suppressed chemical corrosion enabled by the organic–inorganic film caused by the dual additives.

To further confirm the effect of the dual electrolyte additives in suppressing the chemical corrosion and dendrite growth on the Zn anode, the surficial configuration of the Zn anode after 50 cycles at a current density of 1 mA cm^−2^ was characterized by SEM. As shown in Fig. S22a and b,† a mass of vertically growing micro-flakes was observed on the Zn anode after cycling in the blank electrolyte, showing serious dendrite growth. In contrast, in the K-1% (Fig. S22c and d†) and K-1% + Ce-1 electrolytes (Fig. 4g, h, S22e and f†), the Zn anode displayed a dense and flat surface without obvious dendrites or by-products. The composition of the Zn anodes after 50 cycles was also characterized by XRD. As shown in Fig. 4i, obvious diffraction peaks of Zn_4_SO_4_(OH)_6_·4H_2_O by-product were observed on the Zn anode after cycling in the blank electrolyte, indicating serious chemical corrosion occurring in the blank electrolyte. Notably, prominent diffraction peaks of 3Zn(OH)_2_·ZnSO_4_·5H_2_O were detected on the Zn anode after cycling in both the K-1% and K-1% + Ce-1 electrolytes. It should be emphasized that the 3Zn(OH)_2_·ZnSO_4_·5H_2_O shares an identical crystal structure with the *in situ* formed inorganic SEI layer. This similarity implies that the inorganic SEI layer maintains its stability and efficiently restrains the emergence of chemical corrosion throughout the cycling process. To confirm the stability of the SEI layer, XPS analysis of the Zn anode after 50 cycles was conducted. As shown in Fig. S23,† C, Si, and N elements can be obviously observed on the surface of the long-term cycled Zn anode, indicating that the SEI layer has good structural stability and can provide long-term protection to the Zn anode. Thus, it can be firmly established that the as-formed organic–inorganic hybrid SEI layer effectively impedes the formation of dendrite growth and chemical corrosion, thereby conferring the Zn anode with remarkable cycling stability.

### Electrochemical performance of the full cells using the dual electrolyte additives

**Fig. 5:**
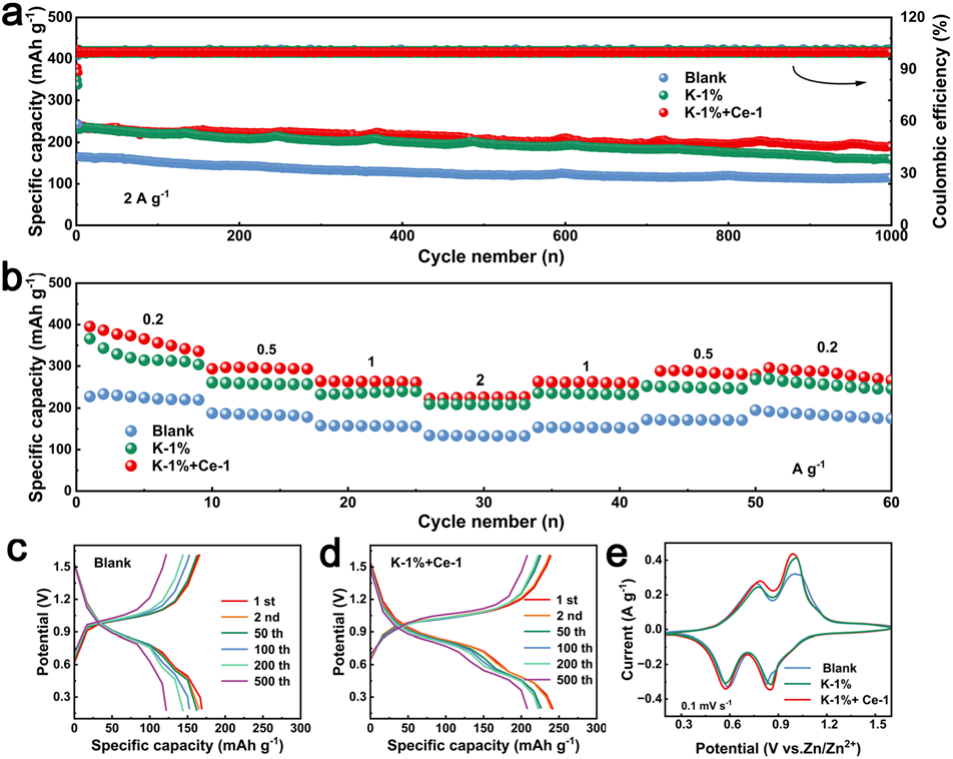
(a) Electrochemical performance of Zn‖V_2_O_5_ cells in different electrolytes at a current density of 2 A g^−1^. (b) Rate performance of Zn‖V_2_O_5_ batteries in different electrolytes at current density ranging from 0.2 A g^−1^ to 2 A g^−1^. The corresponding charge–discharge curves at 2 A g^−1^ in (c) blank electrolyte and (d) K-1% + Ce-1. (e) Cyclic voltammetry curves of the cells at the scan rate of 0.1 mV s^−1^.

Based on the understanding of the role of the dual additives in modifying the Zn anode, the practical application potential was further evaluated by fabricating Zn‖V_2_O_5_ full cells.^59^ As shown in Fig. 5a, the Zn‖V_2_O_5_ cell with K-1% + Ce-1 electrolyte delivers a high initial capacity of 239.46 mA h g^−1^ and maintains 188.35 mA h g^−1^ after 1000 cycles, showing a high capacity retention rate of about 78.7%. The initial capacity obtained in K-1% electrolyte is 231.13 mA h g^−1^, which is also much higher than that obtained in the blank electrolyte (165.09 mA h g^−1^). The excellent electrochemical performance of the full cell in K-1% and K-1% + Ce-1 electrolytes is attributed to the improved stability of the Zn negative electrode. The charge–discharge curves shown in Fig. 5c and d further confirm the good cycling stability of the cells in the optimized electrolytes. The rate performance of the full cells was also measured at the steeply increased current density. As shown in Fig. 5b, the cell with K-1% + Ce-1 electrolyte exhibits average capacities of 365.6, 295.2, 262.7, and 226.4 mA h g^−1^ at current densities of 0.2, 0.5, 1, and 2 A g^−1^, respectively and then maintains 287.9 mA h g^−1^ when the current returns to 0.2 A g^−1^, displaying excellent rate performance and cycling stability. As shown in Fig. 5e, the CV curves of the cell of Zn‖V_2_O_5_ in the three electrolytes are similar in shape, and all of them have two pairs of redox peaks corresponding to the reversible deposition/stripping reaction of Zn^2+^/H^+^ in V_2_O_5_.^60^ Obviously, the peak areas of the K-1% + Ce-1 and K-1% full cells are larger than those of the blank cells, indicating the enhancement of the capacity of the modified cells.

## Conclusions

In summary, dual electrolyte additives based on amino silane and Ce_2_(SO_4_)_3_ were employed to *in situ* construct an organic–inorganic hybrid SEI layer for a stable Zn anode. The hybrid SEI film formed by the adsorption of amino silane-linked Zn hydroxyl sulfate on the surface of the Zn anode achieves homogeneous interfacial ion distribution and induces uniform Zn deposition, thus inhibiting Zn dendrite growth. Moreover, the as-formed SEI layer separates the Zn anode from the electrolyte, thus suppressing the decomposition of water and the HER reaction. Lastly, Ce^3+^ shields the tips of the protuberances and induces uniform Zn deposition. In the K-1% + Ce-1 electrolyte, the Zn anode delivers high reversibility of >5000 h at a current density of 1 mA cm^−2^ with an areal capacity of 1 mA h cm^−2^, as well as a high CE of 99.49%. The Zn‖V_2_O_5_ cells assembled with the dual additives also demonstrate enhanced lifetime and capacity. These results indicate that the application of an amino silane/Ce_2_(SO_4_)_3_ dual electrolyte additive provides a practical and effective strategy for developing high-performance ZIBs.

## Data availability

The data supporting this article have been included as part of the ESI.†

## Author contributions

Q. W., C. W. and H. M. conceived the idea of this study, L. Y., S. H., B. L. and M. Z. performed the experiments. Q. W. and C. W. supervised the conduct of this study. L. Y. provided the first draft of the paper that was corrected by Q. W. and C. W. All authors critically reviewed the manuscript and approved the final version for submission.

## Conflicts of interest

There are no conflicts to declare.

## Supplementary Material

SC-OLF-D5SC00718F-s001
