# Supplementary material for: Silane cooperation with Ce2(SO4)3 to efficiently construct a protective layer and induce uniform deposition of Zn2+ for an ultra-stable Zn anode
Source: Chem Sci. 2025 Mar 26;16(19):8319–26. doi: 10.1039/d5sc00718f (PMC11979620; doi:10.1039/d5sc00718f)
Supplement: SC-016-D5SC00718F-s001 [file SC-016-D5SC00718F-s001.pdf]

Supplementary Information (SI) for Chemical Science.
This journal is © The Royal Society of Chemistry 2025

Supporting Information

Silane cooperation with $Ce_2(SO_4)_3$ to efficiently construct a protective layer and induce uniform deposition of Zn^{2+} for an ultra-stable Zn anode

Luyan Yu,[a] Sidan He,[a] Baohua Liu,[a] Mingrui Zhang,[a] Houyi Ma,[b] Chao Wang,*[a] and Qinghong Wang,*[a]

a. *School of Chemistry and Materials Science, Jiangsu Normal University, Xuzhou, Jiangsu 221116, P. R. China.*

E-mail: wangc@jsnu.edu.cn (C. Wang), wangqh@jsnu.edu.cn (Q. Wang)

b. *School of Chemistry and Chemical Engineering, Shandong University, Jinan, Shandong 250100, P.R. China*

Materials Characterization:

The interactions between KH-540 and Zn^{2+} were measured by FTIR (Japan-Shimadzu IRTracer 100) and XPS (America-Thermo Fisher Scientific K-Alpha+ spectrometer equipped with a monochromatic Al Kα X-ray source (1486.6 eV) operating at 100 W). The Zn anode morphology was observed by scanning electron microscopy (SEM, Japan Hitachi SU-8010). The side reaction on Zn anode was characterized by XRD (German Bruker, D8 ADVANCE). Structure of zinc flake surface analyzed by atomic force microscopy (AFM, German Bruker Dimension Icon). The contact angle tester (German Dataphysis-OCA20) tested the degree of wetting of the electrolyte with the zinc negative electrode.

Preparation of the electrolyte and electrode

The electrolytes of mono amino silanes were prepared by dissolving 2 M $ZnSO_4$ in deionized water, adding different volume fractions of KH-540 to a blank electrolyte and stirring at a stirring rate of 1200 r min^{-1} for 5 min, followed by ultrasonication for 3 min to obtain a homogeneously dispersed electrolyte. The aqueous solutions of 2 M $ZnSO_4$ electrolyte containing volume fractions of 0.5 vol %, 1 vol %, and 2 vol % KH-540 additives are referred to as K-X (X = 0.5, 1, and 2 %). The additive package was prepared by dissolving 2 M $ZnSO_4$ in deionized water, adding different concentrations of cerium sulfate, and ultrasonicating it for 10 min to dissolve it. KH-540 was then added to the above solution and prepared as in the case of a single amino silane electrolyte. Aqueous solutions of 2 M $ZnSO_4$ electrolyte containing 1 vol % KH-540 and Ce^{3+} concentrations of 0.5-, 1-, and 5-mM additives are referred to as K-X%+Ce-Y (X=0.5, 1, and 2, Y=0.5, 1, and 5 mM). Zn foil was pressed into sheets of 16 mm diameter and used as Zn negative electrode. The positive electrode was prepared by mixing 70 wt % V_2O_5, 20 wt % Super P and 10 wt % polytetrafluoroethylene (PTFE), and then using the rolling method, resulting in an area loading of about 1 mg cm^{-2} of the prepared V_2O_5.

Electrochemical measurements:

Zn||Cu asymmetric cells, Zn||Zn symmetric cells and the full cells in various electrolytes were assembled using CR2032 coin-type cells and their electrochemical properties were evaluated on the NEWARE CT-2001A battery test systems (Shenzhen, China). In these cells, anode zinc foils and cathode zinc foils have diameters of 16 mm and 10 mm, respectively, and thicknesses of 0.1 mm, with an electrolyte loading of 80 μL per cell. Cyclic voltammetry (CV), the differential capacitance, linear sweep voltammetry (LSV), electrochemical impedance spectroscopy (EIS), chronoamperometry (CA), Tafel plot tests were carried out on a CHI660E electrochemical workstation to investigate the electrochemical behavior of the cells. All the electrochemical tests were conducted at room temperature (25 °C). LSV was measured by using a tri-electrode system, using Pt as the counter electrode and Ag/AgCl as the reference electrode, at a scan rate of 10 mV s^{-1} and a voltage range of -1 to -1.4 V. Tafel plots were measured by using a tri-electrode system, using Pt as the counter electrode and Ag/AgCl as the

reference electrode, at a scan rate of 1 mV s^{-1} over a voltage interval between -0.8 and -1.1 V. EIS measurements were conducted on an electrochemical workstation (CHI 660E) over a frequency ranging from 100 kHz to 10 mHz. The CA measurement was examined on Zn||Zn symmetric cells by applying a constant overpotential of -150 mV for 400 s. CV tests were measured using a Zn||V_2O_5 cell assembled using a button cell abrasive (760YP-24B) with a scan rate of 0.1 mV s^{-1} and a voltage range of 0.2 to 1.6 V. Differential capacitance was measured on a Solartron 1287 frequency analyzer using a Zn||Zn button cell within a voltage range of 10 mV, -0.4 to 0.4 V sweep rate.

The transference number of Zn^{2+} ($t_{Zn^{2+}}$) was calculated from the EIS and CA data of a Zn||Zn symmetric cells through the following formula:

$$t_{Zn^{2+}} = \frac{I_S(\Delta V - I_0 R_0)}{I_0(\Delta V - I_S R_S)} \quad (1)$$

Where I_0 and R_0 are the initial current and resistance, Is and Rs are the steady-state current and resistance after polarization, ΔV is the polarization voltage (25 mV).

The activation energy (Ea) was calculated from the EIS data (using CHI900D) of symmetric batteries at the temperature gradient through the following formula:

$$\frac{1}{R_{ct}} = Aexp(\frac{E_a}{RT}) \quad (2)$$

Where Rct is the Zn^{2+} charge transfer resistance; R is the molar gas constant; T is the Kelvin temperature; A is the pre-exponential factor.

Measurement of the Ionic Conductivity of the SEI Layer:

$$\sigma = \frac{l}{R \cdot A} \quad (3)$$

The impedance spectra of the SEI layer-modified stainless-steel electrodes were collected at 25 °C in a frequency ranging from 100 kHz to 10 mHz to determine the ionic conductivity of the organic-inorganic solid/electrolyte interface layer. The ionic conductivity (σ) was estimated based on Equation where R is the resistance, A is the area, and l is the thickness of the SEI layer

Adsorption of Ce^{3+} on Zn plane (002) slab were calculated with the CP2K package version-2022.1 [S1] using Gaussian Plane Wave (GPW) method implemented in the QUICKSTEP module [S2]. Perdew-Burke-Ernzerhof (PBE) [S3] exchange-correlation (XC) functional with Grimme-D3 [S4, S5] dispersion correction method was employed. Both the double-zeta valence polarized (DZVP) sets and Goedecker-Teter-Hutter (GTH) pseudopotentials were adopted [S6-S8]. Plane wave and relative cut-offs were set to 400 and 55 Ry, respectively. The inner and outer SCF convergence criteria were set to 2.0×10^{-6} Ha. To avoid interactions between periodic images, a vacuum distance of 30 Å was imposed between different layers. The geometrical optimizations were implemented at the *Γ* point for all surface structures. The bottom two layers of atoms were frozen while the top two were allowed to relax. Root mean square and maximum force convergence were set to 3.0×10^{-4} and 4.5×10^{-4} Ha·Å^{-1}, respectively.

Figures

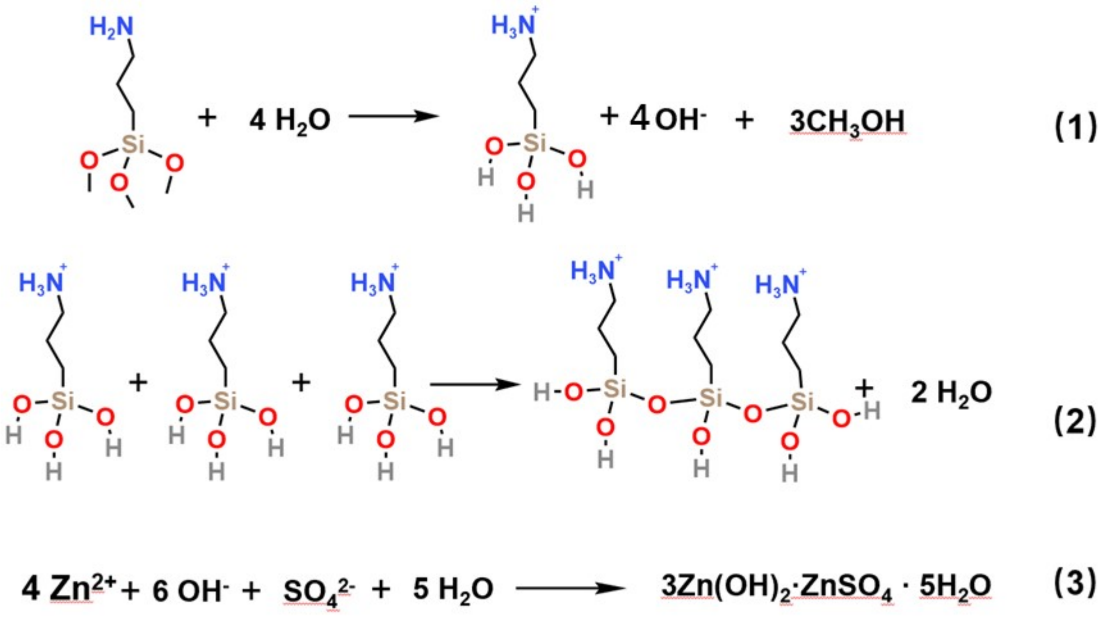

Fig. S1 Reaction formulas of KH-540 in 2M $ZnSO_4$.

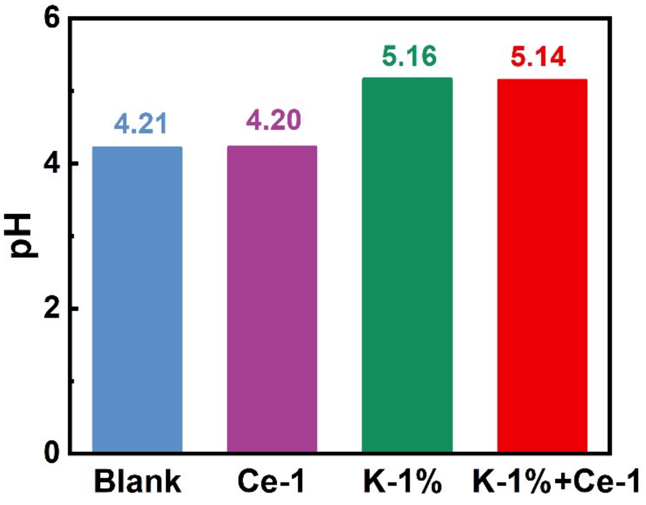

Fig. S2 The pH values of the electrolytes with KH-540 and/or $Ce_2(SO_4)_3$.

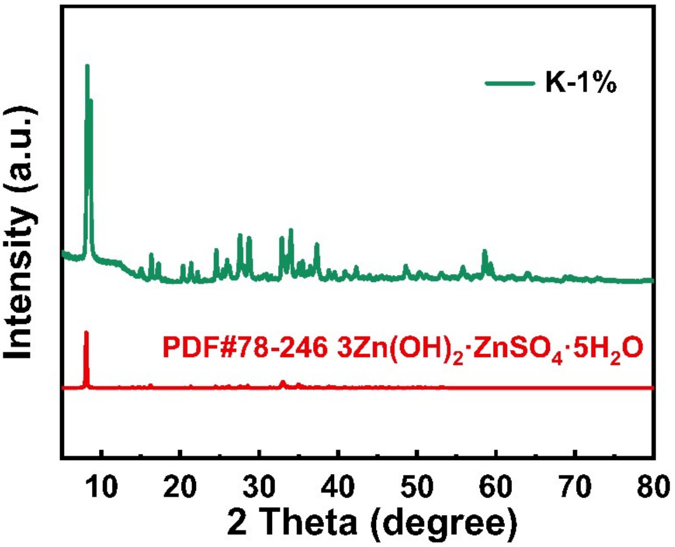

Fig. S3 XRD pattern of the white precipitate produced in the K-1%/$ZnSO_4$ solution.

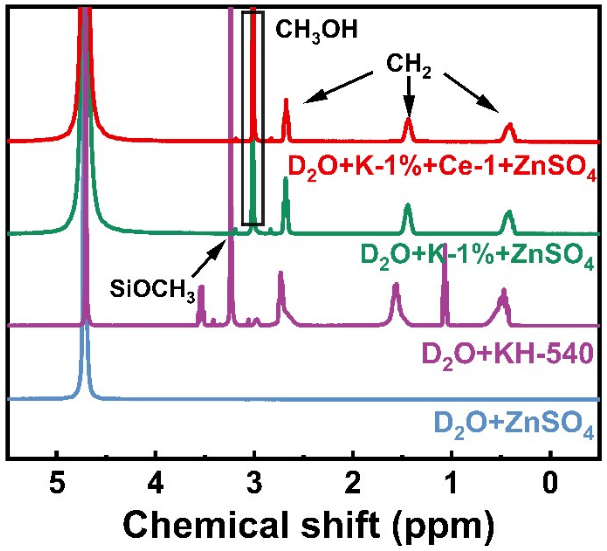

Fig. S4 ^{1}H NMR spectra of 2 M $ZnSO_4$, KH-540 (1%), K-1%/$ZnSO_4$ and K-1%+Ce-1/$ZnSO_4$ solutions.

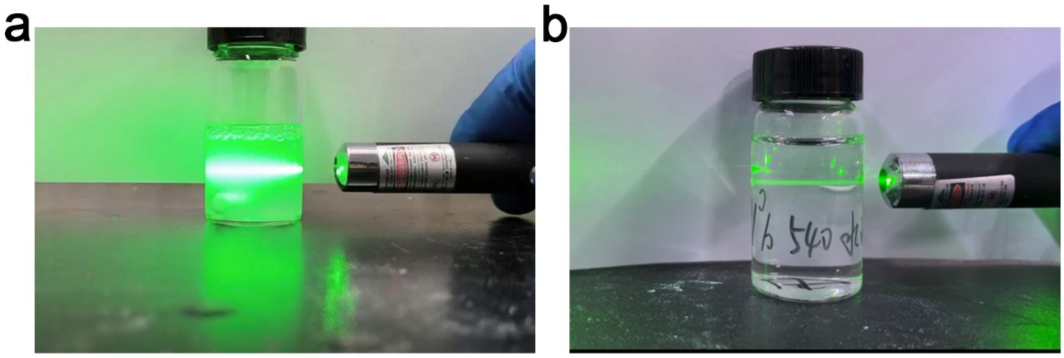

Fig. S5 Tyndall effect test for the (a) K-1%/$ZnSO_4$ solution and (b) K-1%/H_2O solution.

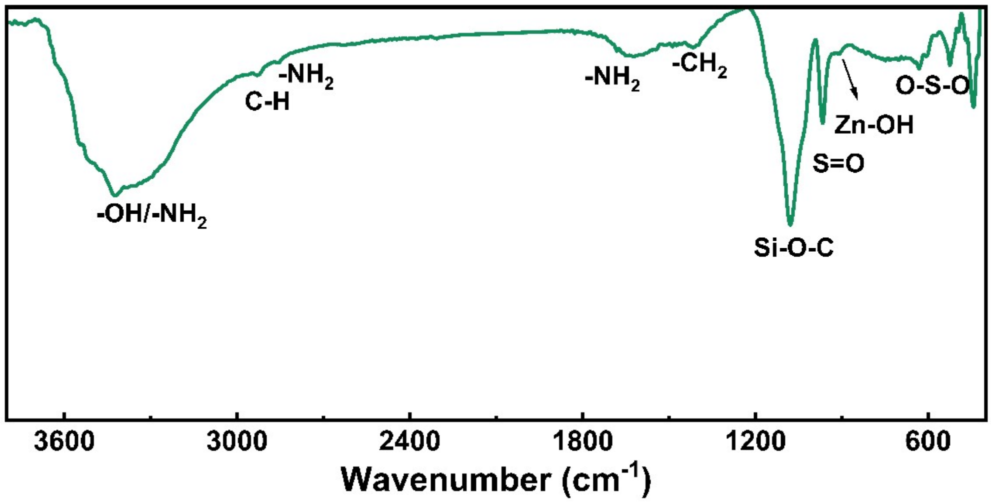

Fig. S6 FTIR spectrum of white precipitate produced in the K-1%.

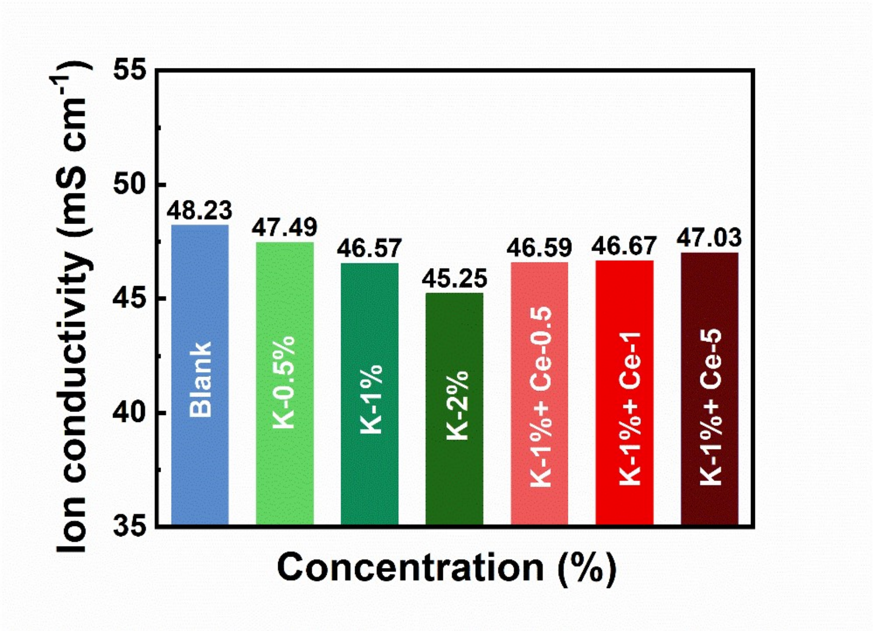

Fig. S7 The ionic conductivity of the electrolytes with different concentrations of KH-540 and $Ce_2(SO_4)_3$.

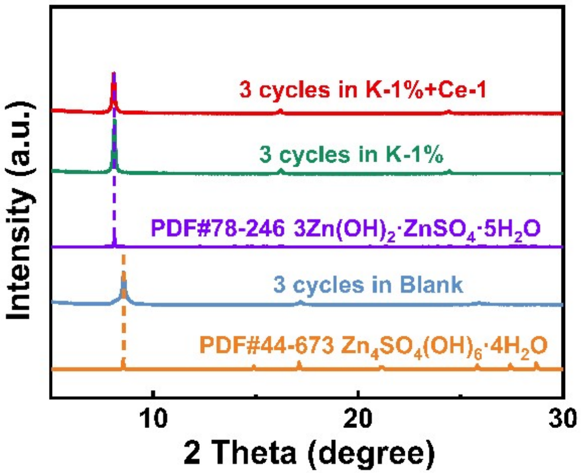

Fig. S8 XRD pattern of the Zn anode after 3 cycles in different electrolytes.

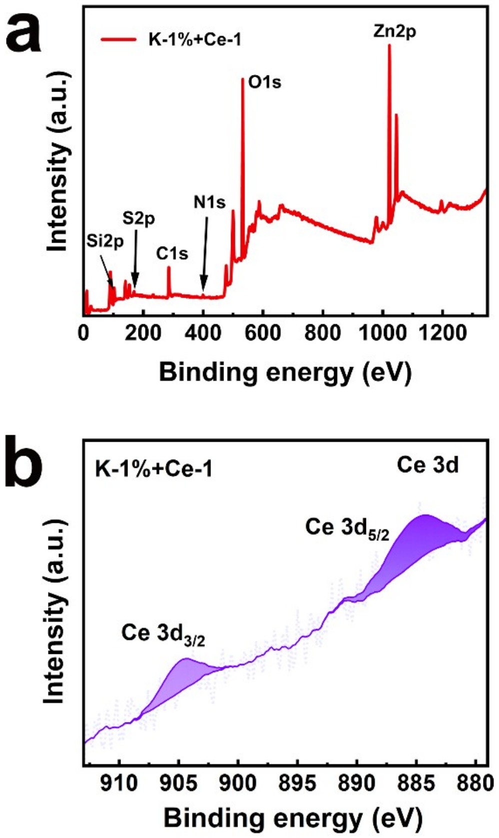

Fig. S9 (a) XPS spectra of the Zn anode surface after 3 cycles in K-1%+Ce-1 and (b) the high-resolution XPS spectra of Ce 3d.

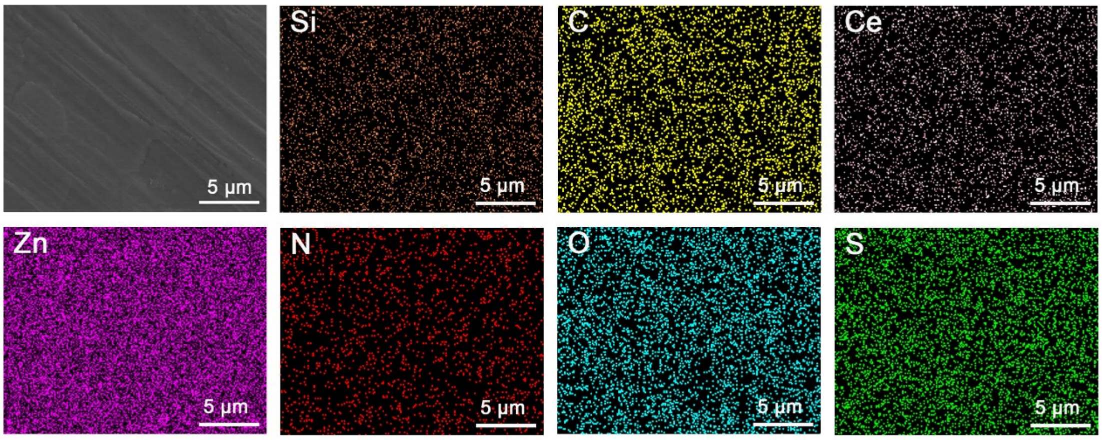

Fig. S10 SEM image and the corresponding EDS mapping of the Zn anode after 3 cycles in K-1%+Ce-1.

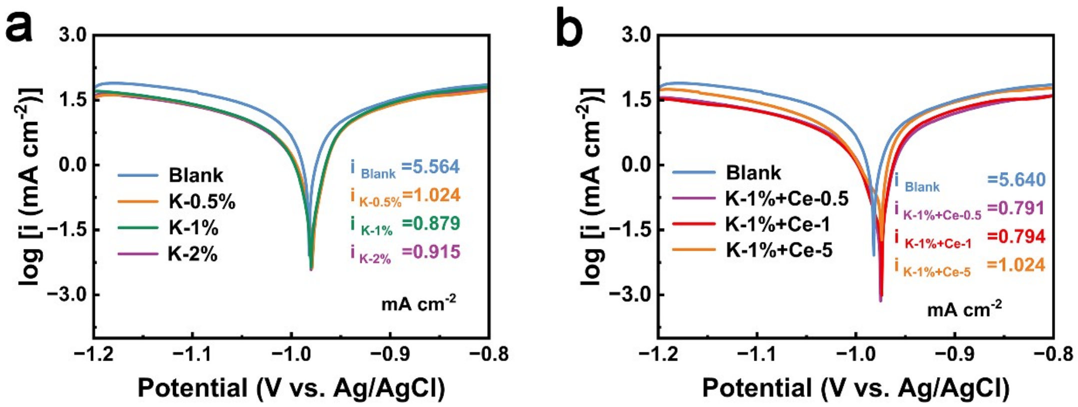

Fig. S11 Tafel polarization plots of Zn anodes in each electrolyte with different amounts of (a) KH-540 and (b) K-1%+$Ce_2(SO_4)_3$.

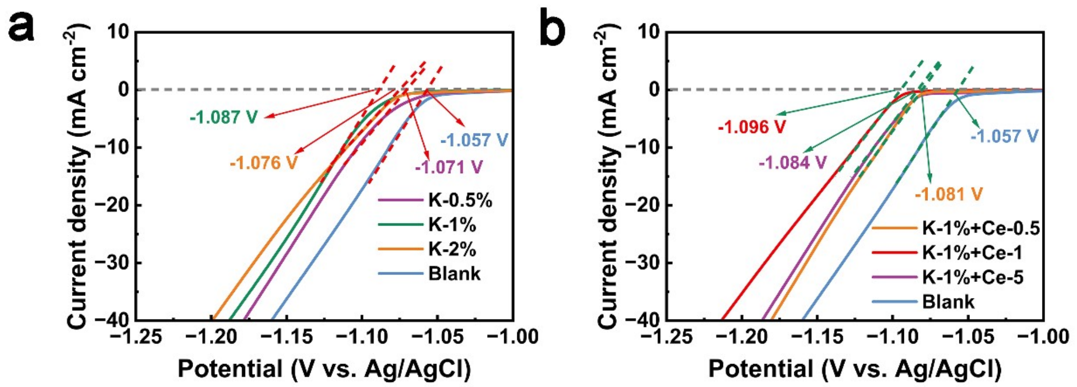

Fig. S12 Linear polarization curves showing the HER potential of Zn anodes in each electrolyte with different amounts of (a) KH-540 and (b) K-1%+$Ce_2(SO_4)_3$.

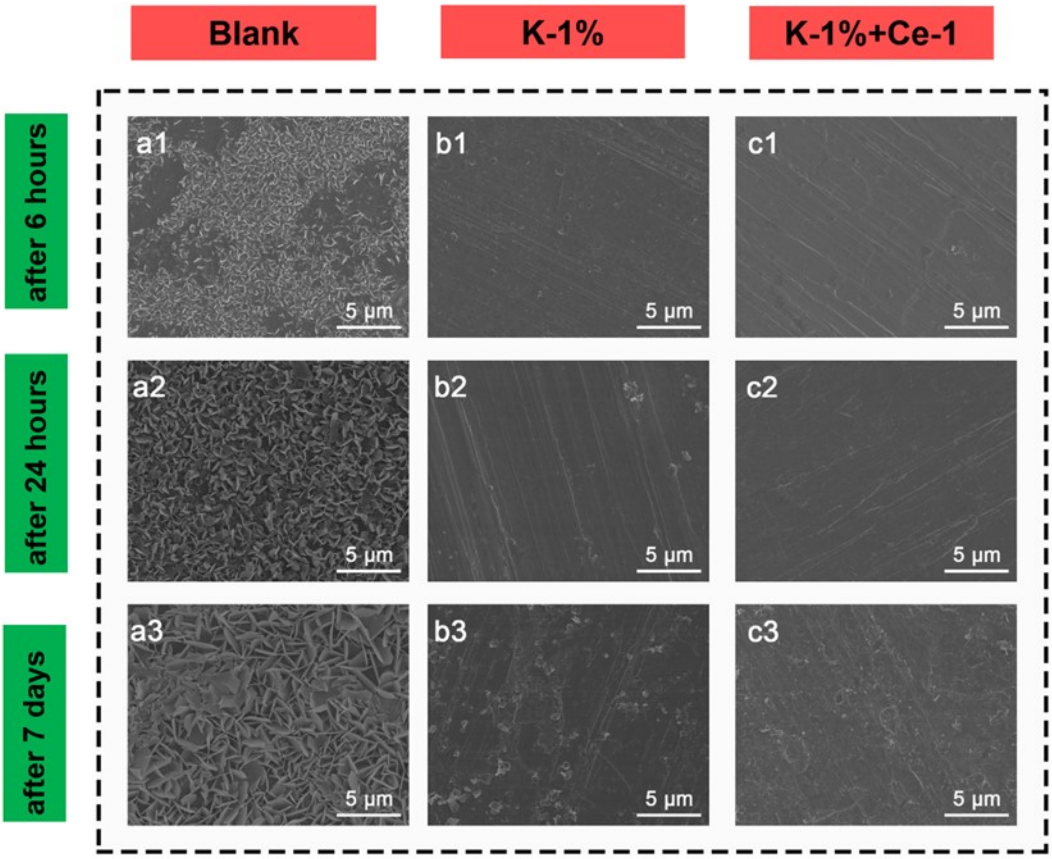

Fig. S13 SEM images for Zn anodes surface after immersing in (a1-a3) blank electrolyte, (b1-b3) the K-1% electrolyte and (c1-c3) the K-1%+Ce-1 electrolyte for different times.

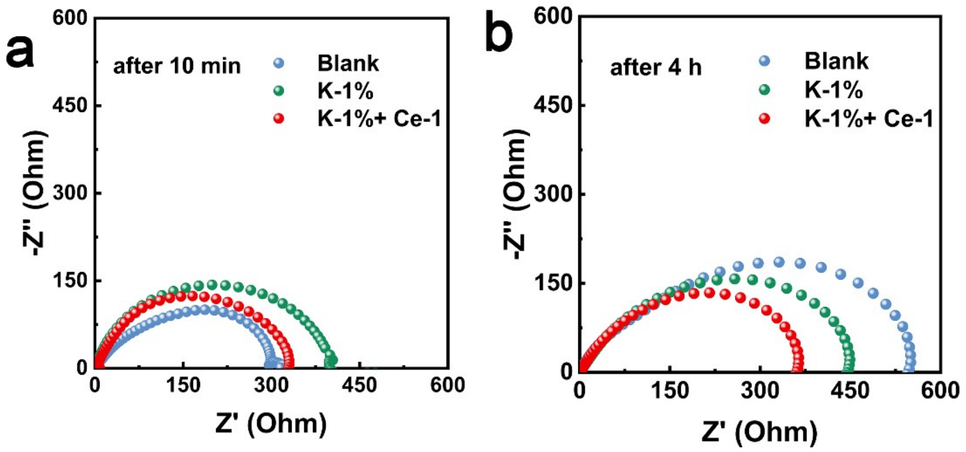

Fig. S14 Nyquist plots of Zn||Zn symmetric cells with different electrolytes after resting for (a) 10 minutes and (b) 4 hours, respectively.

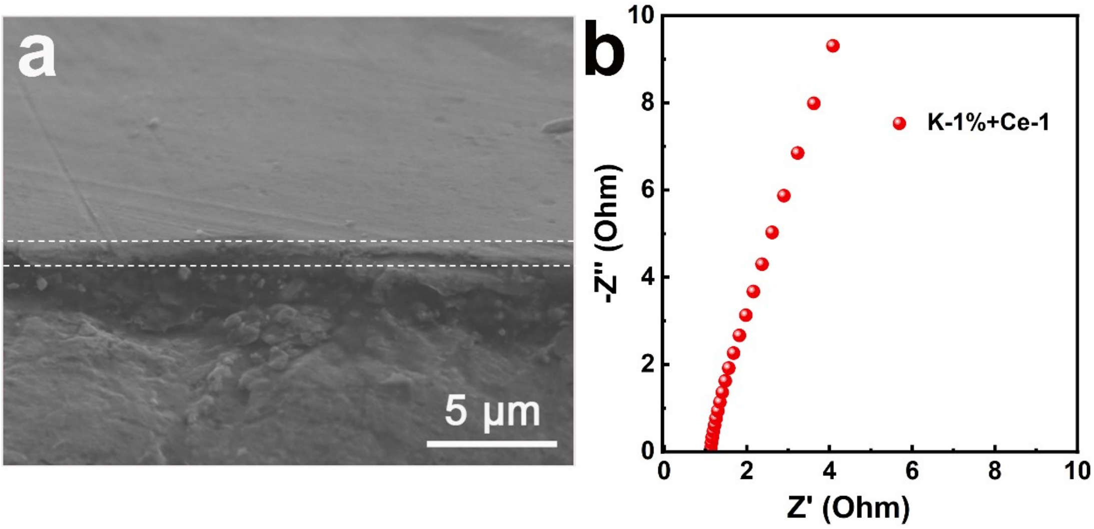

Fig. S15 Side-view SEM image and EIS measurement of the Zn anode after 3 cycles in K-1%+Ce-1 electrolyte.

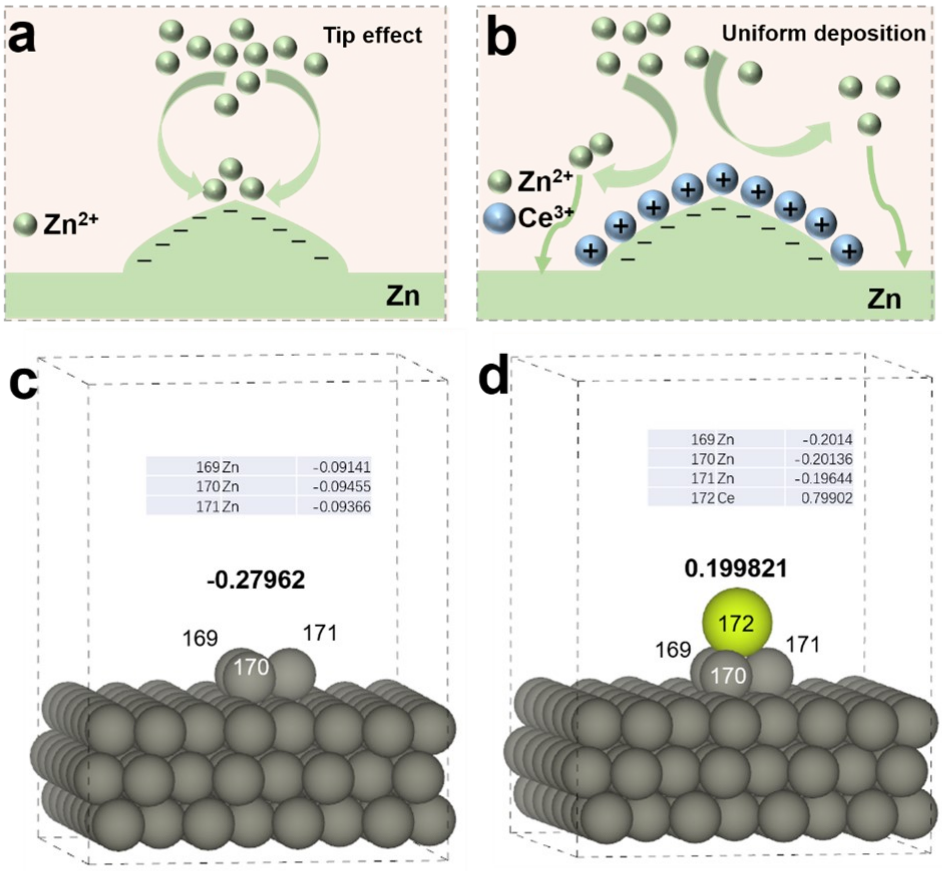

Fig. S16 (a) Schematic diagram of tip effect; (b) Schematic diagram of shielding effect; DFT calculations of the local charge of the protuberance on Zn anode (c) without and (d) with the adsorption of Ce^{3+}.

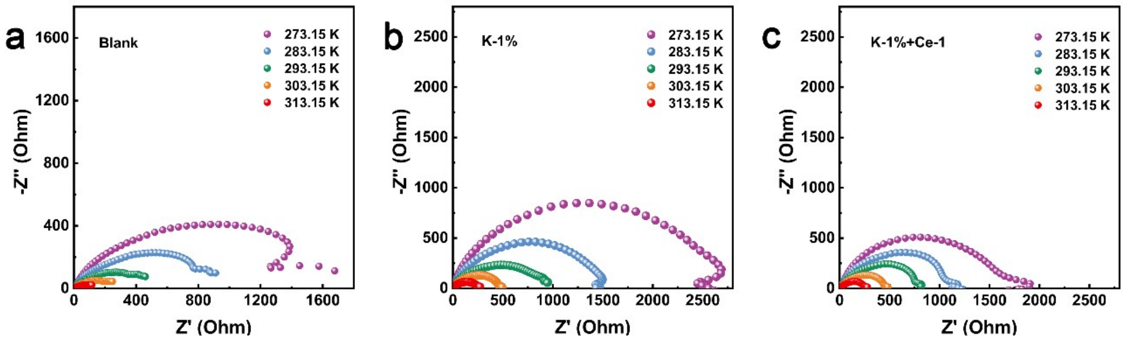

Fig. S17 Nyquist plots of Zn||Zn symmetric cells tested under various temperatures in (a) the blank electrolyte, (b) the K-1% electrolyte and (c) the K-1%+Ce-1 electrolyte.

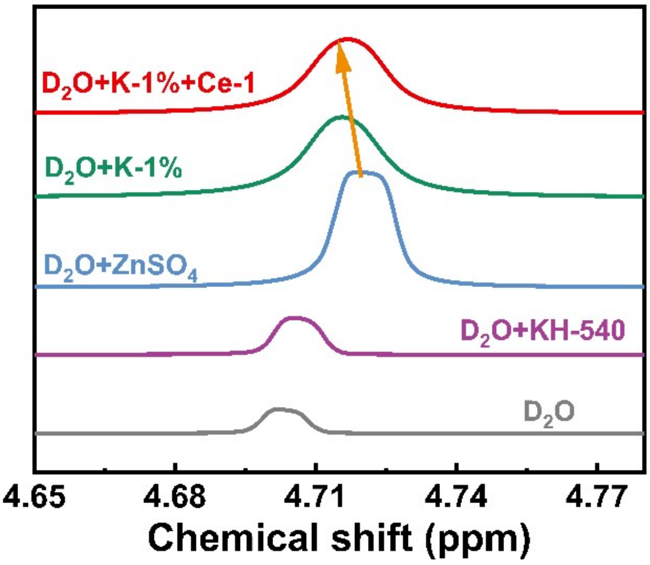

Fig. S18 ^{1}H NMR spectra of different solutions.

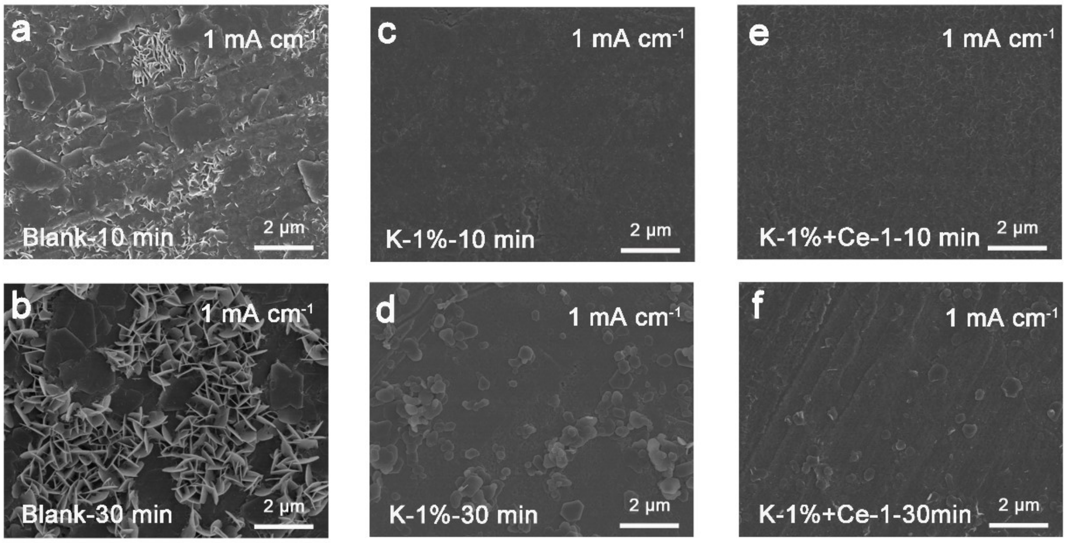

Fig. S19 SEM images of the surface morphology after 10 min and 30 min of deposition at the current density of 1.0 mA cm^{-2} in (a, b) blank electrolyte, (c, d) the K-1% electrolyte and (e, f) the K-1%+Ce-1 electrolyte.

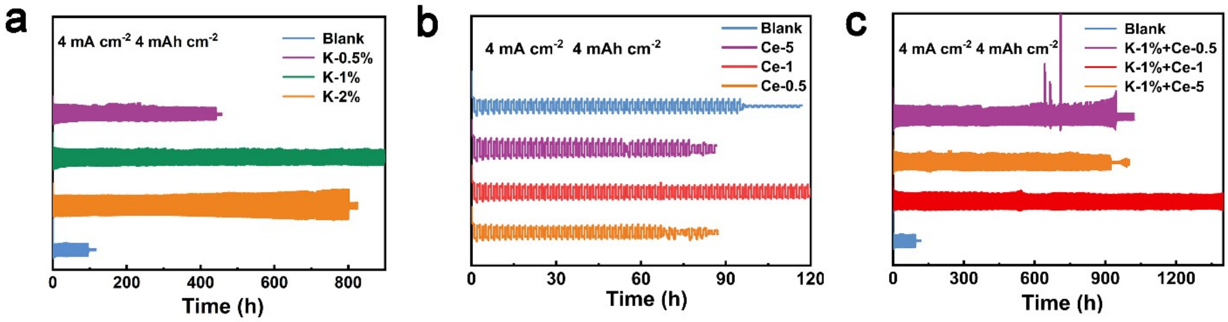

Fig. S20 Long-term cycling performance of Zn||Zn symmetric cells in different electrolytes at the current density of 4 mA cm^{-2} with the areal capacity of 4 mAh cm^{-2}.

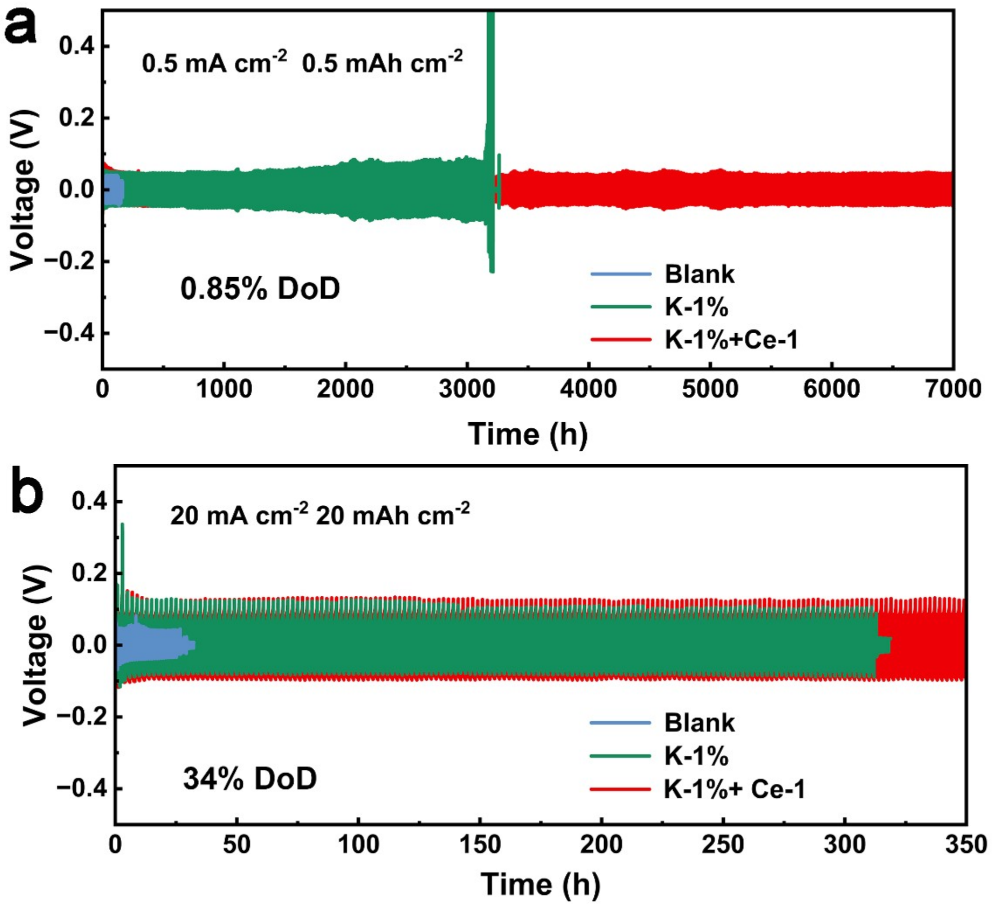

Fig. S21 Long-term cycling performance of Zn||Zn symmetric cells in different electrolytes at (a) 0.5 mA cm^{-2} and 0.5 mAh cm^{-2} and (b) 20 mA cm^{-2} and 20 mAh cm^{-2}.

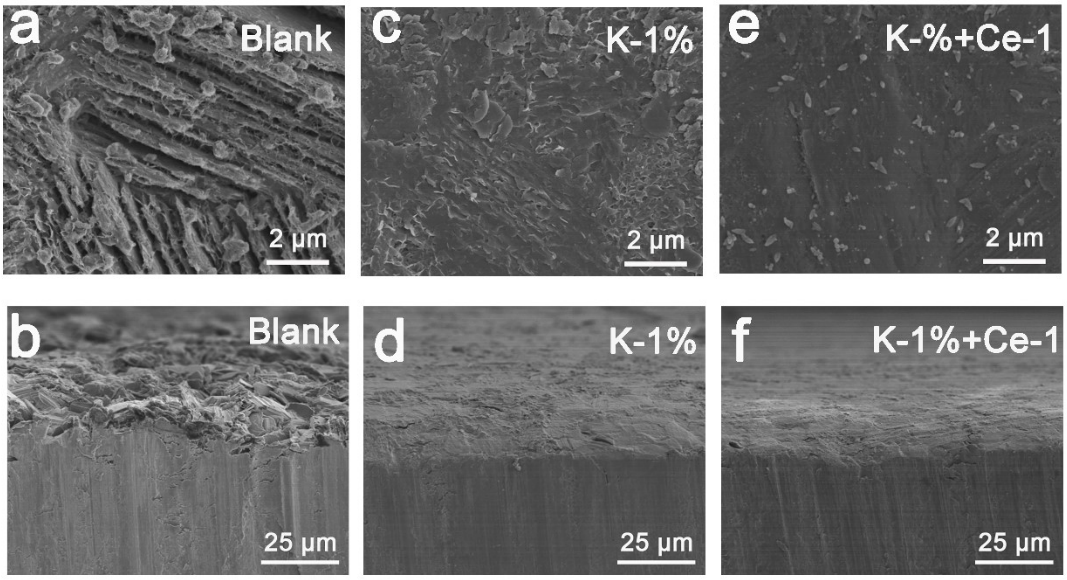

Fig. S22 SEM images of Zn anode surface after 50 cycles at a current density of 1 mA cm^{-2} in (a, b) blank electrolyte, (c, d) the K-1% electrolyte and (e, f) the K-1%+Ce-1 electrolyte.

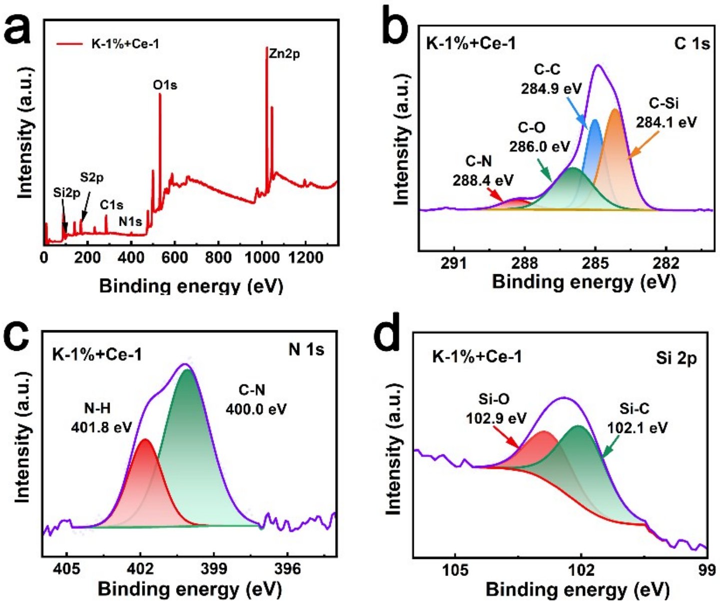

Fig. S23 (a)XPS spectra of the Zn anode after 50 cycles in K-1%+Ce-1 electrolyte, and the high-resolution XPS spectrum of (b) C 1s, (c) N 1s, (d) Si 2p.

Table S1. Comparison of cycle life of the cells using K-1%+Ce-1 electrolyte with the reported electrolytes modified with different additives.

**Modified electrolyte with different additives**	**Performance of symmetric cells Current (mA cm^{-2})/Areal capacity (mAh cm^{-2})/Cycle life (h) DOD/Cycle life (h)**	**References**
$TiOSO_4$ + 2 M $ZnSO_4$	1 / 1 / 3750<br>5 / 1 / 1000<br>10 / 1 / 2300	Ref. S9
D-Arabinose (DA) + 2 M $ZnSO_4$	1 / 1 / 950<br>2 / 1/ 700<br>10 / 10 / 400	Ref. S10
Lactobionic acid (LA) + 2 M $ZnSO_4$	1 / 1 / 2300<br>10 / 10 / 800	Ref. S11
Triethyl phosphate (TEP) + 2 M $ZnSO_4$	0.5 / 0.5 / 1500<br>1 / 1 / 5000<br>5 / 5 / 700	Ref. S12
phytic acid (PA) + 1 M $ZnSO_4$	1 / 1 / 1200<br>2 / 1 / 1000<br>5 / 1 / 400	Ref. S13
Glutamate(NCAP) + 2 M $ZnSO_4$	1 / 1 / 2000	Ref. S14
Potassium citrate + 2 M $ZnSO_4$	0.5 / 0.5 / 2000<br>2 / 2 / 1000<br>10 / 2.5 / 1600	Ref. S15
(3-(1-Pyridinio)-1-propanesulfonate（PPS）+ 2 M $ZnSO_4$	1 / 1 / 5700<br>2 / 2 / 2400<br>10 / 10 / 450<br>50 / 5 / 500	Ref. S16
1,3-Dimethyl-2-imidazolidinone + 2 M $ZnSO_4$	2 / 1 / 1600<br>10 / 10 / 900	Ref. S17
Tranexamic acid (TXA) + 2 M $ZnSO_4$	1 / 1 / 2100<br>5 / 5 / 700	Ref. S18
KH540+Ce^{3+} + 2 M $ZnSO_4$	0.5 / 0.5 / 7000<br>1 / 1 /5000<br>4 / 4 1400<br>10 / 10 900<br>30 / 30 / 300	This work

References

[S1] T. D. Kühne, M. Iannuzzi, M. Del Ben, V. V. Rybkin, P. Seewald, F. Stein, T. Laino, R. Z. Khaliullin, O. Schütt, F. Schiffmann, D. Golze, J. Wilhelm, S. Chulkov, M. H. Bani-Hashemian, V. Weber, U. Borštnik, M. Taillefumier, A. S. Jakobovits, A. Lazzaro, H. Pabst, T. Müller, R. Schade, M. Guidon, S. Andermatt, N. Holmberg, G. K. Schenter, A. Hehn, A. Bussy, F. Belleflamme, G. Tabacchi, A. Glöß, M. Lass, I. Bethune, C. J. Mundy, C. Plessl, M. Watkins, J. VandeVondele, M. Krack and J. Hutter, CP2K: An electronic structure and molecular dynamics software package - Quickstep: Efficient and accurate electronic structure calculations, *J. Chem. Phys.*, 2020, **152**, 194103.

[S2] J. VandeVondele, M. Krack, F. Mohamed, M. Parrinello, T. Chassaing and J. Hutter, Quickstep: Fast and accurate density functional calculations using a mixed Gaussian and plane waves approach, *CoPhC*, 2005, **167**, 103-128.

[S3] J. P. Perdew, K. Burke and M. Ernzerhof, Generalized Gradient Approximation Made Simple, *PhRvL*, 1996, **77**, 3865-3868.

[S4] S. Grimme, J. Antony, S. Ehrlich and H. Krieg, A consistent and accurate ab initio parametrization of density functional dispersion correction (DFT-D) for the 94 elements H-Pu, *J. Chem. Phys.*, 2010, **132**, 154104.

[S5] S. Grimme, S. Ehrlich and L. Goerigk, Effect of the damping function in dispersion corrected density functional theory, *JCoCh*, 2011, **32**, 1456-1465.

[S6] J. VandeVondele and J. Hutter, Gaussian basis sets for accurate calculations on molecular systems in gas and condensed phases, *J. Chem. Phys.*, 2007, **127**, 114105.

[S7] S. Goedecker, M. Teter and J. Hutter, Separable dual-space Gaussian pseudopotentials, *PhRvB*, 1996, **54**, 1703-1710.

[S8] C. Hartwigsen, S. Goedecker and J. Hutter, Relativistic separable dual-space Gaussian pseudopotentials from H to Rn, *PhRvB*, 1998, **58**, 3641-3662.

[S9] J. Zhang, C. Zhou, Y. Xie, Q. Nan, Y. Gao, F. Li, P. Rao, J. Li, X. Tian and X. Shi, Inorganic Electrolyte Additive Promoting the Interfacial Stability for Durable Zn-Ion Batteries, *Small*, 2024, **20**, 2404237.

[S10] Y. Yang, Y. Li, Q. Zhu and B. Xu, Optimal Molecular Configuration of Electrolyte Additives Enabling Stabilization of Zinc Anodes, *Adv. Funct. Mater.*, 2024, **34**, 2316371.

[S11] R. Jiang, T. Naren, Y. Chen, Z. Chen, C. Zhang, Y. Xie, L. Chen, Y. Qi, Q. Meng, W. Wei and L. Zhou, Enhanced Hydrogen Bonding Through Strong Water-Locking Additives for Long-Term Cycling of Zinc Ion Batteries, *Adv. Funct. Mater.*, 2024, **34**, 2411477.

[S12] P. Wang, H. Zhou, Y. Zhong, X. Sui, G. Sun and Z. Wang, Dendrite-Free Zn Metal Anodes with Boosted Stability Achieved by Four-in-One Functional Additive in Aqueous Rechargeable Zinc Batteries, *Adv. Energy Mater.*, 2024, **14**, 2401540.

[S13] Y. Chen, F. Gong, W. Deng, H. Zhang and X. Wang, Dual-function electrolyte additive enabling simultaneous electrode interface and coordination environment regulation for zinc-ion batteries, *Energy Stor.Mater.*, 2023, **58**, 20-29.

[S14] X. Fan, L. Chen, Y. Wang, X. Xu, X. Jiao, P. Zhou, Y. Liu, Z. Song and J. Zhou, Selection of Negative Charged Acidic Polar Additives to Regulate Electric Double Layer for Stable Zinc Ion Battery, *Nano-Micro Lett.*, 2024, **16**, 270.

[S15] Y. Wang, H. Gao, C. Guo, F. Chen, D. Jia, M. Xu, L. Ai, N. Guo and L. Wang, Reshaping the Electrolyte Solvation Structure and Electrical Double Layer with Potassium Citrate To Stabilize the Zn Anode, *ACS Appl. Energy Mater.*, 2024, **7**, 10487-10495.

[S16] S. Qin, J. Zhang, M. Xu, P. Xu, J. Zou, J. Li, D. Luo, Y. Zhang, H. Dou and Z. Chen, Formulating Self-Repairing Solid Electrolyte Interface via Dynamic Electric Double Layer for Practical Zinc Ion Batteries, *Angew. Chem. Int. Ed.*, 2024, **63**, e202410422.

[S17] Y. Meng, M. Wang, J. Wang, X. Huang, X. Zhou, M. Sajid, Z. Xie, R. Luo, Z. Zhu, Z. Zhang, N. A. Khan, Y. Wang, Z. Li and W. Chen, Robust bilayer solid electrolyte interphase for Zn electrode with high utilization and efficiency, *Nat. Commun.*, 2024, **15**, 8431.

[S18] J. Yin, H. Liu, P. Li, X. Feng, M. Wang, C. Huang, M. Li, Y. Su, B. Xiao, Y. Cheng and X. Xu, Integrated electrolyte regulation strategy: Trace trifunctional

tranexamic acid additive for highly reversible Zn metal anode and stable aqueous zinc ion battery, *Energy Stor.Mater.*, 2023, **59**, 102800.
